# Dual Chromatic Laser-Printed Microfluidic Paper-Based Analytical Device (μPAD) for the Detection of Atrazine in Water

**DOI:** 10.1021/acsomega.3c04387

**Published:** 2023-10-28

**Authors:** Hichem Moulahoum

**Affiliations:** Biochemistry Department, Faculty of Science, Ege University, Bornova, Izmir 35040, Turkey

## Abstract

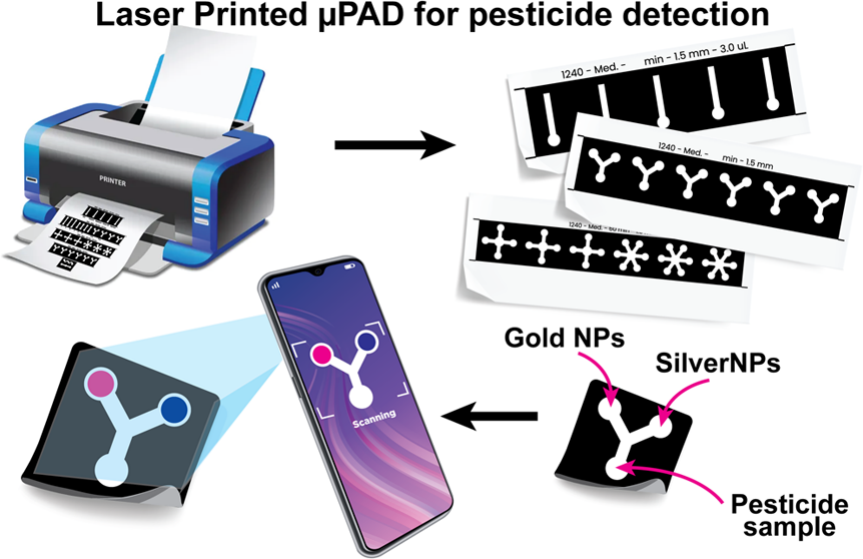

Water pollution caused by pesticides is a significant threat to the environment and human health. Silver and gold nanoparticle (AgNPs, AuNPs)-based biosensors are affordable tools, ideal for environmental monitoring. Microfluidic paper-based devices (μPADs) are a promising approach for on-site testing, but few studies have explored the use of laser printing (LP) for μPAD-based biosensors. This study investigates the feasibility of using laser printing to fabricate paper-based biosensors for pesticide detection in water samples. The μPAD was designed and optimized by using different filter paper porosities, patterns, and channel thicknesses. The developed LP-μPAD was used to sense the pesticide atrazine in water through colorimetric assessments using a smartphone-assisted image analysis. The analytical assessment showed a limit of detection (LOD) of 3.5 and 10.9 μM for AgNPs and AuNPs, respectively. The sensor had high repeatability and reproducibility. The LP-μPAD also demonstrated good recovery and functionality in simulated contaminated water. Furthermore, the detection of pesticides was found to be specific under the influence of interferents, such as NaCl and pH levels. By combining laser printing and nanoparticles, the proposed sensor could contribute to developing effective and low-cost solutions for monitoring water quality that are widely accessible.

## Introduction

Water is an essential resource for life, and its quality is vital for both the environment and human health. However, water pollution is a widespread problem that threatens the availability and quality of this vital resource.^1^ Pesticides are one of the main sources of water pollution as they are widely used in agriculture to protect crops from pests. Once applied, pesticides can enter water bodies through various pathways such as runoff and leaching. Their persistence, toxicity, and bioaccumulation potential significantly threaten aquatic ecosystems and human health.^2^ Developing efficient approaches is crucial to detect and monitor pesticide contamination in water.

Traditional analytical methods for pesticide detection, such as gas and liquid chromatography, involve pricey equipment, experienced staff, and lengthy procedures.^3−5^ Moreover, these methods are unsuitable for on-site monitoring and may require sample pretreatment or specialized sample containers. In contrast, small-factor and portable biosensors have gained tremendous popularity and attention in various applications, including environmental monitoring. They can be qualitative and quantitative, made from different materials (e.g., paper, plastic, glass), and provide different types of signals (e.g., electrochemical, colorimetric).^6−8^

Signal molecules are important components in developing paper-based colorimetric assays, which are widely used in environmental monitoring due to their simplicity, rapid response, and low cost. These assays detect a color change from a specific reaction between the target analyte and a signal molecule.^9−11^ Nanoparticles, including gold and silver nanoparticles, have been extensively used as signal molecules due to their unique optical properties and easy synthesis.^12^ They offer various possibilities for developing paper-based colorimetric assays with enhanced sensitivity, specificity, and multiplexing capabilities for environmental monitoring applications.

Analytical chemistry has witnessed a growing interest in developing microanalytical devices suitable for point-of-care (POC) and point-of-need applications in environmental monitoring. Recent advancements in this area involve using microfluidic paper-based analytical devices (μPADs), which employ paper as a substrate for creating microfluidic channels by incorporating hydrophobic barriers.^13,14^ This innovative approach presents numerous advantages over traditional analytical devices made from glass, silicon, and polymers. These advantages include cost-effectiveness, disposability, ease of fabrication, portability, and the absence of the need for pumps. These distinctive features make μPADs particularly appealing for POC testing in resource-limited and less-industrialized regions. Several reports have demonstrated the efficacy of μPADs in detecting a broad range of analytes, yielding promising results.^15−18^

Researchers have developed different techniques to create microfluidic paper-based analytical devices (μPADs), including laser etching, photolithography, and plasma treatment. Nonetheless, these methods have certain disadvantages such as requiring costly equipment and time-consuming fabrication processes. This may hinder the widespread use and manufacture of μPADs in regions with limited resources and developing countries. Additionally, the operation and maintenance of such equipment require skilled personnel, adding to their production cost.^19−21^ The high cost of equipment and technical expertise required makes them impractical in resource-limited settings. As such, efforts to develop cost-effective and user-friendly techniques are being pursued in research to enhance the accessibility of μPADs for more comprehensive applications.

One promising approach to fabricating and mass-producing μPADs is printing techniques that deposit digitally designed patterns onto paper substrates. Several printing techniques are available, such as inkjet and wax printing.^19−22^ Wax printing is a popular technique that involves printing a wax pattern onto a paper substrate, which creates hydrophobic barriers that define microfluidic channels. Inkjet printing, on the other hand, uses ink droplets to print patterns directly onto a paper substrate. Both techniques have advantages and limitations regarding resolution, versatility, and scalability. Wax printing uses relatively costly printers, whereas inkjet printing uses various solvents that can damage the cartridge over time.^19−22^ Therefore, the printing feasibility still needs further enhancement to be applicable in resource-limited settings.

Laser printing is a promising technique that uses a laser beam to selectively remove material from a substrate, creating high-resolution patterns. This technique is flexible and adaptable to a broad spectrum of substrates, encompassing paper, plastic, and metal. Moreover, laser printing offers high precision, reproducibility, and scalability, making it a promising candidate for the mass production of paper-based microfluidic biosensors.^21,23,24^ Despite its potential, few studies have explored laser printing for μPAD-based biosensors.^23,24^

Taken together, the objectives of this study address two main ideas. First, we aim to investigate the feasibility of using laser printing to fabricate paper-based microfluidic biosensors for pesticide detection in water samples. We will evaluate the performance of laser-printed microfluidic channels in terms of precision, reproducibility, and sensitivity, aiming to provide insights into the potential of laser printing for paper-based microfluidic biosensors in environmental analysis. Second, we will explore the use of nanoparticles in the presence of their pesticide target under simulated contaminated water to develop a robust and versatile platform for pesticide detection in environmental samples. The combination of laser printing and nanoparticles could contribute to the development of effective and low-cost solutions for monitoring water quality and protecting human health and the environment.

## Experimental Section

### Materials and Equipment

Two types of quantitative filter papers of Ø125 cm (FILTER-LAB, FILTROS ANOIA, S.A., Barcelona, Spain) with different porosities (#1240 with a pore size of 14–18 μm and #1238 with 20–25 μm pores) were used to fabricate the laser-printed μPADs (LP-μPADs). Food-grade red color dye (Ponceau 4R) was obtained from Karakas Boya Baharat Kimyevi Maddeler SAN. Ve TIC. Ltd. STI (Istanbul, Turkey). Gold nanoparticles (AuNPs) were purchased from Sigma-Aldrich (Germany). A monochrome laser printer (Canon LBP2900 Laser Shot) with an original toner cartridge (Crg-703) available in our office was used. Other chemicals were of analytical grade and used without additional purifications.

### Synthesis of Blue-Colored Citrate-AgNPs

Citrate-AgNPs were synthesized using sodium borohydride (NaBH_4_), hydrazine sulfate, and trisodium citrate as a reducing agent, coreductant/stabilizer, and stabilizer, respectively.^25^ Briefly, 30 mL of trisodium citrate (30 mM), 20 mL of NaBH_4_ (3.0 mM), and hydrazine sulfate (2.0 mM) were mixed for 20 min at room temperature. Silver nitrate solution (5.0 mL, 3.0 mM) was gradually added to the preprepared solution, causing color change until reaching the blue color, indicating the formation of citrate-AgNPs. The particles were characterized using DLS, SEM, and UV–vis following standard protocols.

### Fabrication of LP-μPADs

**Figure 1:**
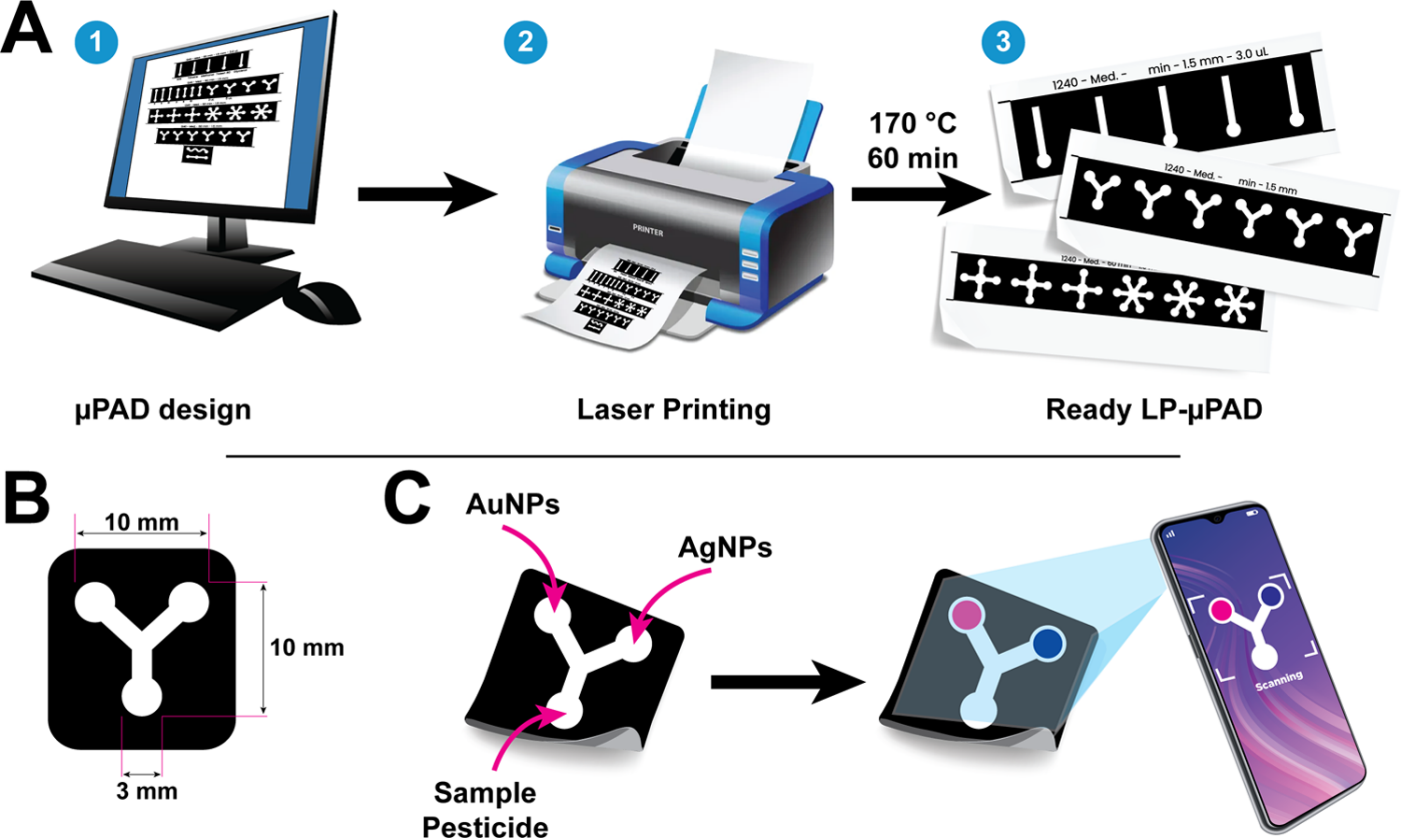
Design, preparation, and analysis steps of the developed LP-μPAD. (A) Preparation of the LP-μPAD in three simple steps, including design of the pattern in the computer (1), printing over filter paper using a laser printer (2), and baking the printed paper to let the ink penetrate (3). (B) Dimensions of the selected pattern used for sensing in the current study. (C) Simplified demonstration of the biosensor preparation for detection using both AgNPs and AuNPs followed by the deposition of the pesticide sample and analysis of the color change using a smartphone camera and ImageJ program.

Creating the LP-μPAD involves several steps, as illustrated in Figure 1A. A pattern design was initially meticulously prepared by using Adobe Illustrator software (V2019). Subsequently, a laser printer was utilized to print a hydrophobic barrier onto filter paper with the highest printing quality (600 dpi). The printed filter papers were uniformly heated in an incubator (W.C. Heraeus, GmbH, Hanau, Germany) at 170 °C for 30 min to ensure proper paper impregnation with toner ink. This heating process resulted in the formation of a hydrophobic barrier on the paper. After these steps were completed, the LP-μPADs were ready for use.

### Optimization and Characterization of the LP-μPAD

The LP-μPAD preparation was optimized before application. The type of filter paper’s porosity was tested by printing patterns on two papers (#1238 labeled as Fast and #1240 labeled as medium). The baking temperature and time were also optimized by testing different temperatures (160, 170, and 180 °C) at various times (0, 5, 15, 30, 60, and 90 min). After each preparation, a drop of food-grade dye was dropped, and the movement of the sample through the channel was followed. This also allowed for the analysis of the liquid confinement ability in the printed pattern. The features of the laser-printed surface were analyzed under a light microscope (Zeiss Primo Star, Carl Zeiss Microscopy GmbH, Germany). The resolution of the proposed LP-μPAD was assessed by printing different channels of various widths (0.1, 0.2, 0.4, 0.6, 0.8, 1.0, 1.5, and 2.0 mm). Subsequently, the reservoir section of the manufactured LP-μPADs was used to introduce a drop of the dye solution. The movement of color was then observed on the front and back sides of the LP-μPADs. The sample volume was also optimized according to the pattern (3.0 and 5.0 μL). The experiments were performed in triplicate (*n* = 3).

### Colorimetric Analysis of Pesticides Using LP-μPAD

The colorimetric analysis using LP-μPAD was performed by following the optimized conditions established earlier. The LP-μPAD was designed as a “Y” shape, with the bottom being the sample reservoir and the two upper portions being test regions (with AgNPs and AuNPs). The overall size of the μPAD was 10 mm, the channel width was 1.5 mm, and the reservoir’s diameter was set at 3.0 mm (Figure 1B). Briefly, AgNPs and AuNPs (2.0 μL) were deposited in the test regions and left to dry (5 min at room temperature). Afterward, atrazine samples (3.0 μL) at different concentrations (0, 10, 15, 20, 25, 30 mM) were dropped over the sample region and left to move through the μPAD until color development was observed over the AgNPs and AuNPs (20–30 s). For analysis, a smartphone camera was employed to capture images, and using ImageJ software, the RGB values of the regions of interest were extracted from the images (Figure 1C). These RGB (red, green, blue) values were then converted into gray values using the formula gray value = R × 0.299 + G × 0.587 + B × 0.114.^23^ The obtained gray values were utilized to calculate various analytical features of the developed LP-μPAD.^26^ Distilled water served as the negative control for all of the conducted tests.

The applicability of the LP-μPAD for real sample analysis was determined using simulated contaminated water. Briefly, the LP-μPAD sensor was prepared as before. The samples applied were prepared using tap water spiked with the pesticide (10 and 20 μM), and the analysis was conducted as described before, followed by calculating the recovery percentage.

### Analytical Performance Features

The developed LP-μPAD was calibrated by applying different concentrations of atrazine pesticide (0, 10, 15, 20, 25, 30, and 100 μM) and measuring the color intensity. The analysis allows the determination of the detection range and linearity, the limit of detection (LOD = 3 × SD_intercept_/slope), and the limit of quantification (LOQ = 10 × SD_intercept_/slope) with SD meaning standard deviation.^26^

The repeatability and reproducibility of the sensor were estimated using a single concentration of atrazine (10 μM) applied over ten (*n* = 10) LP-μPADs printed and prepared from the same batch (repeatability) and another ten (*n* = 10) LP-μPADs of different batches (reproducibility).

The interferences that might occur during testing over real samples, such as pH and the level of salts, were also explored. Briefly, LP-μPAD was prepared and tested with 10 μM atrazine in the presence of NaCl of various concentrations (0, 10, 15, 20, 25, 30 μM) and buffers of different pHs (tris buffer (pH = 4.6), citrate buffer (pH = 5.4), phosphate buffer (pH = 7.4), and carbonate buffer (pH = 11)). The capacity of the LP-μPAD to detect atrazine under different conditions was estimated as before by analyzing the color intensities. The tests were repeated three times (*n* = 3).

The recovery of LP-μPAD was estimated by applying spiked samples (10 and 20 μM) over the sensor and calculating the concentrations from the established calibration curve (three repetitions). The recovery of the LP-μPAD sensor is expressed in percentages (%) of the spiked sample concentration. The data was also used to calculate the coefficient of variation (CV% = (SD/mean) × 100).

### Statistical Analysis

The results were reported as means ± standard deviations (mean ± SD) derived from multiple experiment repetitions. Statistical analysis was performed using Student’s *t* test and employing GraphPad Prism 8.4.3 software. Statistical significance was considered when the *p*-value was less than 0.05.

## Results and Discussion

The rapid advancement of new technologies and the development of microfluidic systems have revolutionized various fields of science and engineering. In particular, laser printing of μPADs has emerged as a promising technique, with significant implications for diverse applications. One such area of great importance is the detection of pesticides in environmental samples, which is crucial in ensuring food safety and protecting public health. Additionally, the detection of pesticides using colorimetric assays offers numerous advantages, including simplicity, cost-effectiveness, and rapidity.

This work aims to explore the potential of nanoparticle-based colorimetric laser-printed μPADs to detect pesticides, highlighting their environmental and health benefits. The sensor is easily fabricated using basic office and laboratory equipment (i.e., laser printer and filter paper) that allows printing multiple copies of the sensor platform in a single print (sensor size of 10 × 10 mm^2^).

### Synthesis and Characterization of the AgNPs

The synthesized blue AgNPs were prepared and showed an absorption peak at 420 nm (Figure S1A). The synthesized AgNPs were characterized using SEM and dynamic light scattering (DLS), demonstrating spherical and smooth surfaces of 80 nm diameter (Figure S1B,C). It was evident that the synthesized AgNPs were uniformly distributed, with the PDI reaching 0.472. These results are in line with previous reports.^27^ UV–vis spectrometry confirmed the stability of the blue citrate-AgNPs over 7 days, showing none to negligible variations (data not shown) and suggesting their applicability to the subsequent steps. In the current study, AuNPs were commercially available and were used as another chromogenic reporter for the fabrication of LP-μPAD. The use of AuNPs and AgNPs for developing biosensors, especially in detecting pesticides (such as organophosphates), is of common use. Although AuNPs are more preferred due to their colorimetric features and stability, AgNPs are gaining more traction due to the development of various techniques to synthesize colorful AgNPs and enhance their stability.^27−30^

Despite their unique optical properties, metal nanoparticle colloids are inherently unstable and prone to aggregation and precipitation because of their elevated surface energy.^31^ The interaction between metal particles, influenced by attractive or repulsive forces, often leads to collisions and aggregation driven by Brownian motion. Unfortunately, this instability limits their applications, particularly in terms of long-term storage. To address this challenge, it is crucial to stabilize these nanoparticles by capping them with a monolayer molecule during synthesis. Capping agents such as thiols, amines, and phosphines, as well as ligands like small-charged species, macromolecules, and polymers, can significantly enhance the stability of metal nanoparticles.^28,32^

### Fabrication, Characterization, and Optimization of the LP-μPAD

To create microchannels within the LP-μPADs, toner ink from the laser printer generated a hydrophobic barrier over the filter paper. The fabrication procedure consisted of two simple steps: the desired pattern was printed onto the substrate, followed by a heat treatment of the printed paper. This heat treatment resulted in the melting of the ink and its infiltration through the capillary structure of the filter paper, as illustrated in Figure 1A. The hydrophobic barrier created is due to the composition of the employed toner ink cartridge, which includes styrene acrylate copolymer (45–55%), iron oxide (40–50%), and amorphous silica (1–3%).^24,33^ The application of heat caused the components to bind to the capillaries present in the cellulose-based paper, forming a durable and stable hydrophobic barrier.

**Figure 2:**
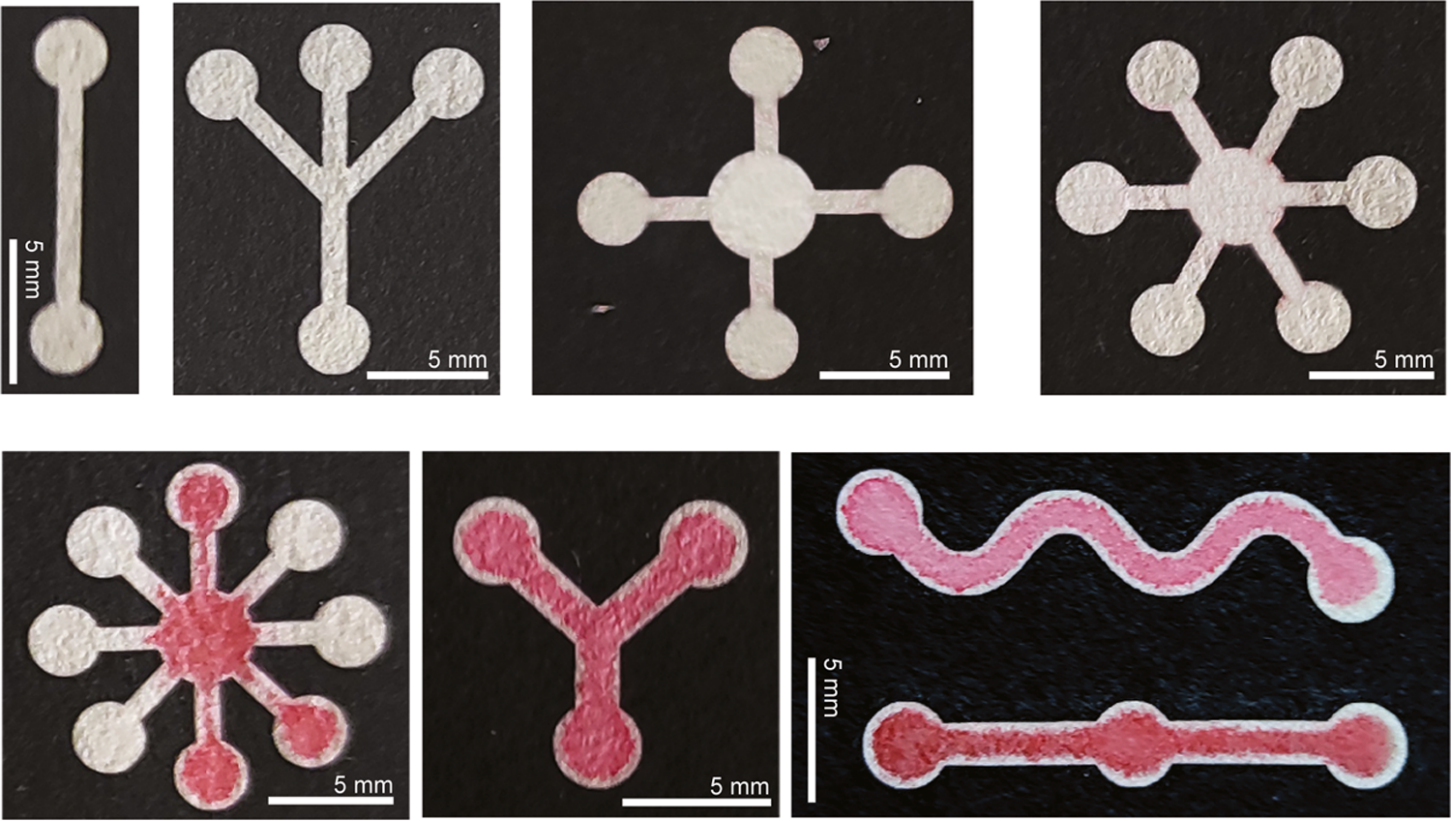
Different patterns printed over filter paper with or without a dye sample show the sample’s ability to move through the generated capillaries (scale bars = 5.0 mm).

To showcase the technique’s flexibility, Figure 2 demonstrates different patterns created on filter paper by using this method. The approach can create several patterns that can be adapted to the application’s needs. The different shapes and widths of the capillary channels need to be coordinated with the number and volumes of samples and reagents. Supporting Movie 1 illustrates the generation of a food dye fluid movement through different patterns designed. The versatility of the proposed technique is highlighted by its capability to create well-defined hydrophobic barriers on various substrates.

In the process of optimizing LP-μPAD, the features of the filter paper were explored. Two different filter papers were selected according to their porosity and labeled as medium (14–18 μm) and fast (20–25 μm) according to the manufacturer. Two different sample volumes were tested (3.0 and 5.0 μL), and the 3.0 μL sample showed better results than the other due to the lower wicking effect through the capillary and random splashing observed (Figure S2A). These volumes are mostly optimized for the tested pattern and should be adjusted for any other pattern employed. At the same time, different heating times were also tested (i.e., 0, 5, 15, 30, 60, and 90 min). The data obtained demonstrated that both filter paper types could be impregnated with the laser printer’s ink. However, the medium filter paper showed better resolution and movement of the tested food dye sample (Figure S2B).

**Figure 3:**
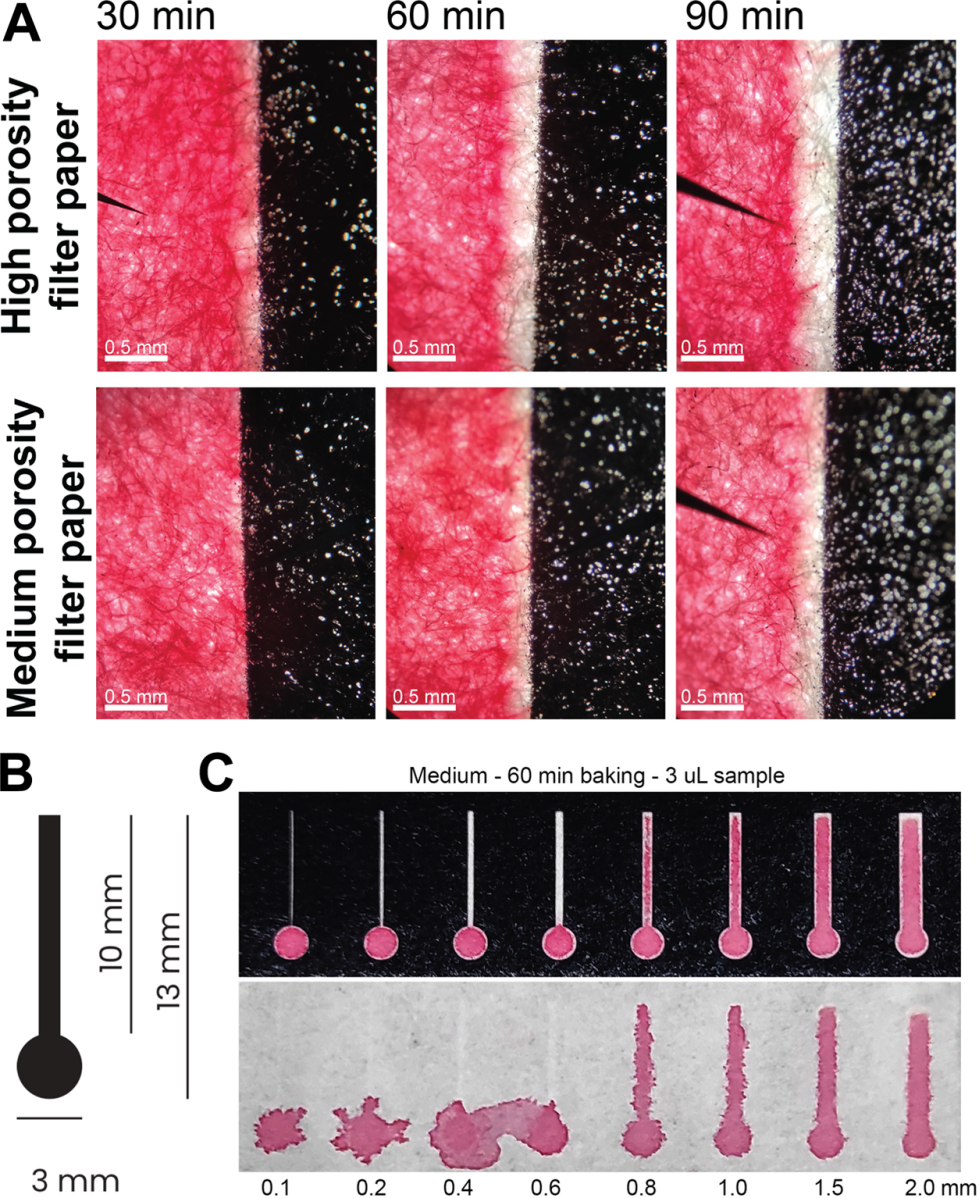
Ink impregnation and capillary movement observation. (A) Analysis of the differences between two types of filter papers patterned and incubated for different times (30, 60, and 90 min) for ink impregnation showing the leaking of dye sample in the 30 min time point while the 60- and 90 min incubation showed a clear creation of the hydrophobic barrier (scale bars = 0.5 mm). (B) Dimensions used for the design of the channels. (C) Front and back observations of the effect of different channel widths on the movement of samples through the LP-μPAD.

The baking time at 0, 5, and 15 min resulted in similar results with random movement of the sample and the creation of splashes in the back of the paper, suggesting that the impregnation was insufficient (data not shown). On the other hand, the baking times of 30, 60, and 90 min presented capillary movement with some differences (Figure S2B). Indeed, the 30 min incubation in the fast filter paper suffered from various setbacks with poor confinement of the liquid through the paper no matter the pattern used or volume deposited, suggesting that the impregnation is still insufficient (Figure 3A). The 60- and 90 min incubation created the hydrophobic barrier, allowing the food dye sample to move through the pattern accordingly (Figure S2). As such, these two incubation times were further explored between the fast and medium filter papers. The results showed that 60 min of incubation produced the most optimal condition for capillary movement and liquid confinement since 90 min of incubation resulted in increased dryness of the filter paper, making the sample move slowly or just stay in place (Figure S2B,C). Upon heating, the particles within the toner ink melt and effectively permeate the cellulosic microfibers in the filter paper. During this process, the black pigment remains concentrated over the surface while the transparent polymeric resin liquefies and penetrates the paper.^24^ The challenges typically encountered with wax-based patterning are effectively addressed by utilizing the solid toner ink method. One of the main difficulties encountered in wax-created hydrophobic barriers is the lack of control over the flow of melted wax through the paper’s pores. This limitation hampers the precise and uniform formation of hydrophobic barriers.^34^ However, employing solid toner ink overcomes this issue, as the ink is more easily controlled and directed during fabrication. This enables a more reliable and consistent formation of hydrophobic barriers within LP-μPADs, ensuring their optimal functionality and performance.

The laser printing process entails applying ink through electrostatic charge interaction among the printing media, toner microparticles, and the photoreceptor drum. This precise technology, commonly found in commercial laser printers, deposits ink over the paper surface, creating hydrophobic regions, thus allowing the construction of high-resolution microfluidic devices. For this study, a laser printer (600 dpi resolution) was employed to ensure the consistent and uniform distribution of toner ink, guaranteeing the reliability and reproducibility of the produced devices.^24^ Within the printer, the ink adheres to the paper’s surface after being partially melted inside the fusion unit. Nevertheless, the brief heating proved inadequate to fully impregnate the ink throughout the paper. Consequently, additional heating is necessary to completely melt the ink components and promote smooth penetration through the cellulose fibers of the filter paper. This additional heating step ensures the proper integration of the hydrophobic ink within the substrate, forming durable and efficient hydrophobic barriers within the LP-μPADs.

Consequently, in addition to the incubation time, the baking temperature was also tested (i.e., 160, 170, and 180 °C), showing that 180 °C has the best results under the current experimental conditions. Our results align with previous reports, where 165–200 °C baking temperatures were considered optimal.^23,24^ These slight differences can be attributed to the differences in the filter paper quality and the type of laser printer’s brand and/or ink composition employed. Indeed, Ghosh et al. used an HP LaserJet Pro printer with an original toner containing 50–55% styrene acrylate resin, 40–45% ferrite, and around 10% wax.^24^

Flow studies were conducted on channels with varying widths ranging from 0.1 to 2.0 mm to assess the proposed platform’s resolving capabilities. Ponceau 4R dye solution was used for these studies, and a total of 10 experiments were performed for each channel width. Different heating durations were employed during the experiments. Results obtained from the investigations revealed that with shorter heating times, the dye exhibited leakage through the channels (Figures 3C and S2). Conversely, with longer heating times, the dye movement was impeded by the lateral diffusion of the polymer into the channel, adversely affecting the resolution (Figure S2). However, it was found that a baking period of 1 h was sufficient to ensure complete penetration of the polymer, thereby producing leak-proof channels (Figure 3).

The experimental observations revealed that a minimum channel width of 0.8 mm was necessary to achieve optimal flow with effective fluid confinement (Figure 3C). It was impossible to fabricate hydrophilic channels with widths less than 0.8 mm because of the filter paper’s thickness constraints. The impregnation of ink throughout the filter paper’s cross section formed a region of around 0.4 ± 0.05 mm, where the polymer wicks toward the hydrophilic channel due to lateral flow induced by the baking process. When creating tight channels (<0.8 mm), it is crucial to consider the decrease in fluid flow speed that accompanies decreasing channel width.^35−37^ Using extremely narrow channels leads to longer fluid travel times due to inverse surface tension effects, ultimately resulting in increased response times.^37^

### Atrazine Sensing via the Dual Chromatic LP- μPAD

In order to enable on-site quantification of pesticides and provide a stable analysis environment, we utilized a smartphone camera for image acquisition in conjunction with the LP-μPAD. The smartphone was positioned atop the LP-μPAD to capture color signals. Our LP-μPAD chromatic sensor, preloaded with AgNPs and AuNPs, successfully detected various concentrations of atrazine under optimal conditions (Figure 1C). The color intensities of both AgNPs and AuNPs gradually changed and deepened with increasing concentrations of atrazine in the standard samples. Subsequently, the RGB values of the images were extracted and converted to gray values for further calculations.

**Figure 4:**
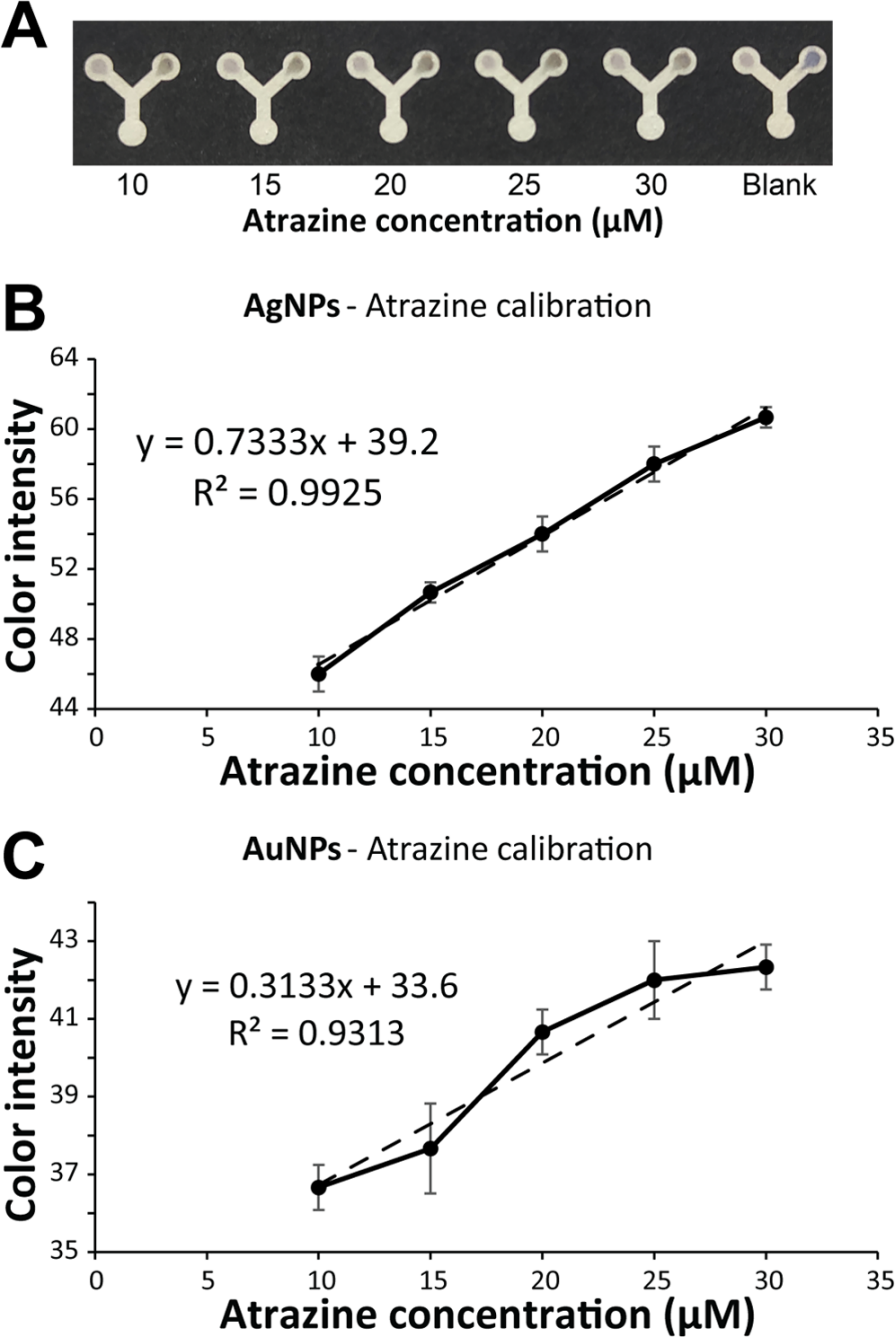
Calibration of the developed LP-μPAD sensor for the detection of atrazine. (A) Visual observation of the developed sensor. (B) Calibration curve of the AgNP-based signals. (C) Calibration curve of the AuNP-based signals.

To determine the detection range of the biosensor, we performed a calibration using increasing concentrations of atrazine ranging from 0 to 100 μM. LP-μPAD exhibited a detection range of 0–30 μM for both AgNPs and AuNPs. We observed good linearity between the color signal and sample concentrations, as evidenced by the regression equations *y* = 0.7333*x* + 39.2 (*R*^2^ = 0.9925) for AgNPs and *y* = 0.3133*x* + 33.6 (*R*^2^ = 0.9313) for AuNPs (Figure 4).

The assay’s corresponding limits of detection (LOD) were determined to be 3.52 and 10.97 μM for AgNPs and AuNPs, respectively. In comparison, the limits of quantification (LOQ) were calculated as 10.67 and 33.26 μM, respectively. Table 1 gives a summary of the analytical features of the developed LP-μPAD.

**Table 1:** Analytical Parameters of the Developed Dual Chromatic LP-μPAD Using AgNPs and AuNPs to Detect Atrazine

	AgNPs-based sensor	AuNP-based sensor
linear range (μM)	10–30	10–30
slope	0.7333	0.3133
S.E. of slope	0.0369	0.0491
intercept	39.2	33.6
S.E. of intercept	0.7831	1.0424
correlation coefficient	0.9924	0.9312
LOD (μM)	3.52	10.97
LOQ (μM)	10.67	33.26
repeatability (CV%)	1.29	1.06
reproducibility (CV%)	1.72	1.55

Combining metal nanoparticles with LP-μPAD and utilizing smartphone imaging and analysis, our approach offers a cost-effective and user-friendly solution for sensing applications. Compared with other detection methods, our method demonstrates clear user-friendliness, accuracy, speed, and portability advantages. Although our data show higher LODs than other developed sensors based on electrochemistry^38,39^ and bioluminescent bacteria,^40^ the current work still performed relatively well compared to other sensors such as cell-free enzymatic sensor with LOD = ∼50 μM,^41^ molecularly imprinted polymer (MIP) potentiometric sensor with LOD = 0.5 μM,^42^ and core–shell nanostructured MIP fluorescent chemosensor with LOD = 1.8 μM.^43^ It is important to note that although various biosensors are being developed, showing various levels of sensitivity and detection range, they need to cover the ranges and limits stipulated by the World Health Organization (WHO) and other countries’ guidelines. The WHO reported that following atrazine application in agricultural areas of many countries, groundwater and drinking -water levels of atrazine were found at levels of 0.01–6 and 0.01–5 μg/L, respectively.^44,45^

### Sensitivity and Interference

**Figure 5:**
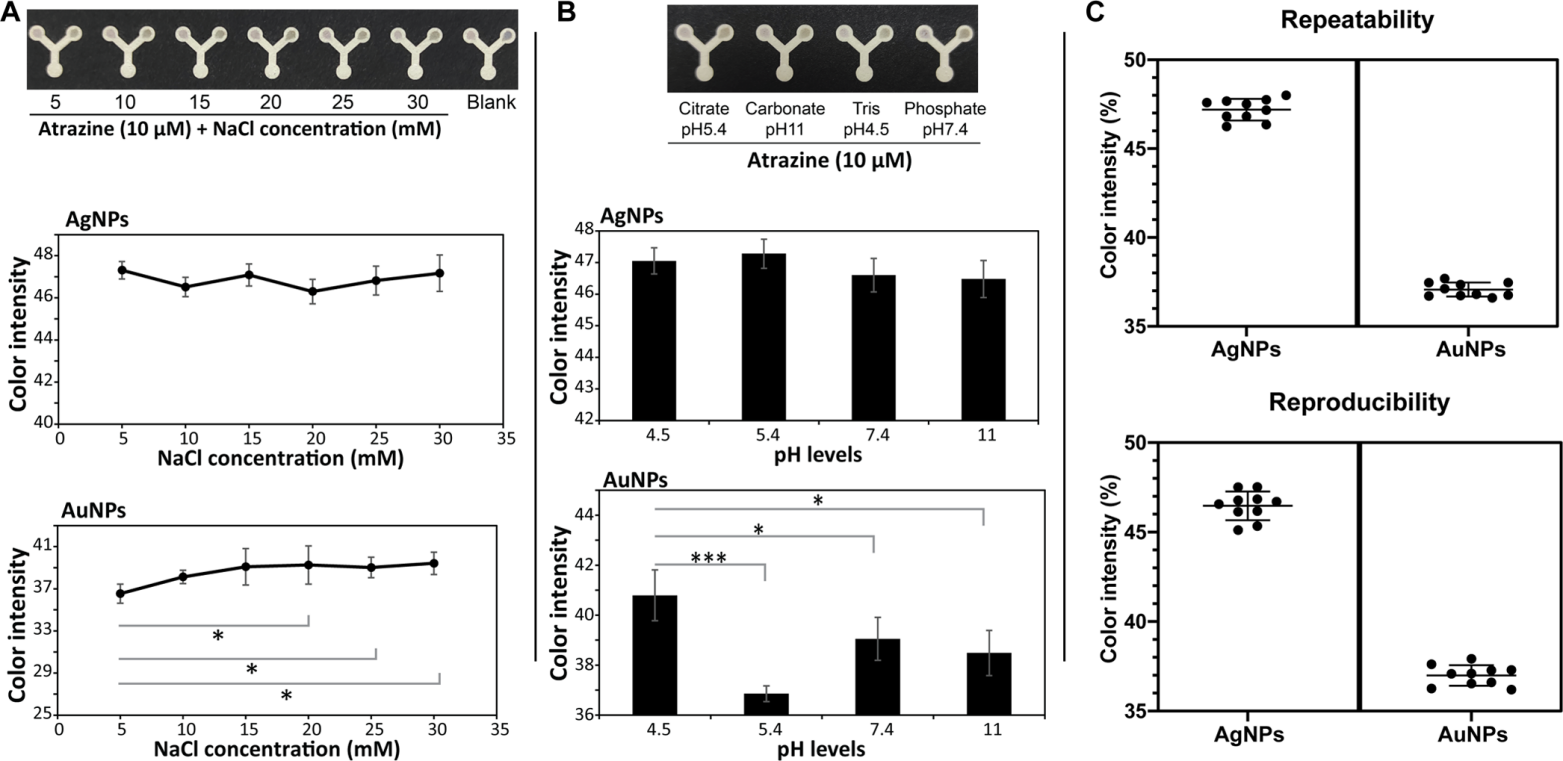
(A, B) Influence of salt concentrations and pH levels on the detection capacity of the LP-μPAD. (C) Repeatability and reproducibility assessments of the developed sensor by repeating the test ten times (*n* = 10) using sensors produced from the same batch (repeatability) and different batches (reproducibility). The sample tested was atrazine at a concentration of 10 μM. ns: non significant; *p < 0.05 and ***p < 0.001.

The study explored the impact of NaCl concentrations ranging from 5.0 to 30 mM on the detection capacity of the developed LP-μPAD sensor. Adding NaCl as an electrolyte can potentially enhance the degree of aggregation of metal nanoparticles, thereby improving the method’s sensitivity. Experimental observations revealed that NaCl concentrations up to 20 mM did not induce any discernible spectral or color changes. However, a slight color shift was observed beyond this concentration, indicating the nanoparticles’ destabilization (Figure 5A). NaCl caused a dose-dependent increase in the color of the gold nanoparticles (AuNPs) due to the aggregation reaction triggered by the salt in the reaction environment. Several studies have documented similar behavior and suggested that the sensor’s functionality is maintained within a certain concentration limit of salts.^46−49^ This observation highlights the importance of considering the salt concentration when utilizing the LP-μPAD sensor and its potential impact on the detection sensitivity.

The colorimetric sensing mechanism employed in this study relies on the aggregation of metallic nanoparticles, specifically silver nanoparticles (AgNPs) and gold nanoparticles (AuNPs), through ligand exchange reactions induced by the presence of pesticides. The pH of the solution plays a key function in the displacement of citrate ligands from the nanoparticle surfaces by the pesticides. To investigate this phenomenon, the effect of pH was examined using four different buffer solutions of different pH values (4.6, 5.4, 7.4, and 11). The results indicated that citrate-stabilized AuNPs exhibited aggregation at lower pH levels (pH = 4.0) due to surface neutralization, resulting in the loss of stabilization (Figure 5B). However, no significant changes in the absorption spectra or color of citrate-stabilized AgNPs were observed in the pH range 4.0 to 11.0. Furthermore, the absorption ratios of AgNPs in the presence of the pesticide were found to be strongly influenced by the solution’s pH. At a buffer pH of 4.0, higher absorption ratios, as indicated by *A*_558_/*A*_405_, were observed, indicating a greater degree of citrate displacement from AgNPs by the pesticide (data not shown). These results indicate that the pesticide exhibits a pronounced tendency to displace citrate ligands from the nanoparticle surfaces under acidic or neutral pH conditions, leading to nanoparticle aggregation. This finding emphasizes the significance of pH control in the colorimetric sensing mechanism and its impact on the detection sensitivity and accuracy of the assay.

### Repeatability and Reproducibility

To assess these parameters, a comprehensive evaluation was conducted by performing repeated tests using μPAD printed from the same paper sheet (repeatability) and several LP-μPAD sets printed in different sheets (reproducibility; Figure 5C). The obtained data demonstrated excellent repeatability for both the AgNPs-based sensor (coefficient of variation (CV) = 1.29%) and the AuNPs-based sensor (CV = 1.06%). Similarly, the reproducibility of the LP-μPAD was found to be highly consistent for both types of nanoparticles, with CV values of 1.72 and 1.55% for AgNPs and AuNPs, respectively (Table 1 and Figure 5C).

The coefficient of variation (CV) is a crucial gauge in assessing the performance of biosensors. The Clinical and Laboratory Standards Institute (CLSI) recommends a CV value lower than 10% for a fabricated sensor to be deemed highly operational.^50^ In the case of the LP-μPAD developed for atrazine detection, the CV values obtained were lower than 2.0% across the various nanoparticles and sensors. This finding further affirms the high analytical capacity of LP-μPAD in providing reliable and accurate data.

### Simulated Contaminated Water

To assess the applicability of the developed strategy in real-world scenarios, we performed the detection of atrazine in spiked tap water samples. The spiked tap water samples were prepared by mixing known concentrations of atrazine (10 and 20 μM) in the tap water matrix and applying it over the LP-μPAD to detect and calculate recovery. The results showed consistent values between the spiked and measured concentrations (Table 2). The recoveries of the two concentrations of atrazine with AgNPs were between 100.2 and 103.4% for both tested concentrations, respectively (CV < 3.5%). These findings contribute to validating and verifying the sensor’s suitability for practical applications, particularly in environmental monitoring and water quality assessment. On the other hand, the AuNP-based sensor side had recoveries ranging between 117 and 122% (CV < 1.7%), suggesting a slight overestimation of the sample. This variation could be attributed to the other components found in water, such as salts and trace metals, as discussed earlier.

**Table 2:** Demonstration of the Developed LP-μPAD’s Sensing Capacity for Detecting Atrazine under Simulated Contaminated Water^a^

	spiked concentration (μM)	measured concentration (μM)	recovery (%)	CV (%)
AgNPs	10	10.02 ± 0.31	100.23 ± 3.14	3.14
	20	20.79 ± 0.29	103.94 ± 1.47	1.42
AuNPs	10	11.79 ± 0.18	117.88 ± 1.89	1.61
	20	24.48 ± 0.28	122.41 ± 1.43	1.17

a Tap water was used, and the mixture was spiked with two different concentrations of atrazine (10 and 20 μM).

## Limitations and Future Directions

While our study showcases the immense potential of LP-μPADs, some limitations must be acknowledged. The current LOD values for atrazine detection, although promising, may not meet the stringent detection requirements of certain regulatory guidelines. Addressing this limitation requires further optimization of the sensing components and integration of the amplification strategies to achieve lower LODs. Additionally, while our dual chromatic approach offers advantages, it may not be applicable to all target analytes, necessitating the exploration of alternative detection schemes for diverse applications. The practical applicability of LP-μPADs in real-world complex matrices, such as natural water samples with varying interferences, warrants further investigation to ensure their reliable performance in the presence of potential interferents.

Another limitation stems from the unavailability of real contaminated water samples and the associated complexities involving regulatory and ethical considerations. Therefore, the experiments were designed to simulate real-world conditions by utilizing spiked tap water, emulating the presence of the target analyte in a water matrix. Although real contaminated water samples would have provided advantages, previous research corroborates the use of spiked tap water as a suitable surrogate for real contaminated water models, offering valuable insights into the sensor’s specificity and detection capabilities.^51−54^

Temperature is also considered as a potential influencing factor on the sensor’s response. While specific experiments directly assessing temperature sensitivity were not conducted, all experiments were meticulously carried out under controlled and consistent temperature conditions. Additionally, an exploration of the effect of temperature (room temperature vs 40 °C) on in-plate sensing of the pesticide atrazine using AgNPs revealed no significant difference in sensor performance between the two test conditions (data not shown). Furthermore, literature findings indicated that the materials and components used in the sensor, such as nanoparticles and the paper substrate, exhibit minimal temperature dependence within the typical environmental range.^55^ Consequently, given the controlled laboratory conditions and insights derived from existing literature, it is strongly believed that the temperature’s influence on the sensor’s response is negligible. This finding underscores the reliability and accuracy of the sensor’s results, irrespective of minor temperature fluctuations encountered during testing.

To further advance the field of LP-μPADs, future directions could include exploring additional nanoparticle combinations and optimization strategies to enhance the detection sensitivity and broaden the range of target analytes. Investigating novel materials, surface chemistries, and functionalization techniques may also lead to improved specificity and selectivity for detection of multiple contaminants simultaneously. Moreover, incorporating additional smartphone-based data processing algorithms and automation features can elevate the usability of the device and its potential for widespread adoption.

## Conclusions

In conclusion, our research has established that laser-printed μPADs are a new and up-and-coming technology with significant analytical and economic potential. Our study’s results have demonstrated the versatility and adaptability of these devices across various applications. Notably, we have successfully fabricated laser-printed μPADs using simple office and laboratory tools, making them easily accessible and cost-effective. Furthermore, the integration of smartphone imaging has proven valuable, enabling on-site applications and real-time data analysis. This capability enhances the practicality and efficiency of LP- μPADs for various fields of application. This work provided an example application focused on detecting atrazine, a commonly used pesticide, using AgNPs and AuNPs as signal molecules. The dual chromatic detection approach yielded promising results, with limit of detection (LOD) values of 3.5 and 10.9 μM, respectively. This demonstrates the high sensitivity and potential of LP- μPADs for pesticide detection in environmental samples.

The economic nature of laser-printed μPADs, combined with their scalability and reproducibility, opens up possibilities for mass production and widespread adoption of this technology. Their simplicity and accessibility make them particularly suitable for resource-limited settings with scarce advanced analytical equipment. With further development and refinement, LP-μPADs have the potential to revolutionize the field of sensing and contribute to sustainable practices on a global scale.

## Supporting Information Available

The characterization of the synthesized AgNPs with regards to UV–vis, SEM, and DLS (Figure S1). The optimization process of the LP-μPAD covering the volume of sample, baking time, filter paper porosity, and channel width (Figure S2) (PDF)
Time-lapse video demonstrating the movement of fluid samples through the patterned channels (Supporting Video 1) (MP4)
