# Supplementary material for: Dual Chromatic Laser-Printed Microfluidic Paper-Based Analytical Device (μPAD) for the Detection of Atrazine in Water
Source: ACS Omega. 2023 Oct 28;8(44):41194–203. doi: 10.1021/acsomega.3c04387 (PMC10633824; doi:10.1021/acsomega.3c04387)

Supplementary Materials

Dual chromatic Laser-Printed Microfluidic Paper-based Analytical Device (µPAD) for the Detection of Atrazine in Water

Hichem Moulahoum*

Biochemistry Department, Faculty of Science, Ege University, Bornova, Izmir, Turkey

***Corresponding authors**

hichem.moulahoum@ege.edu.tr (H.M)

Figure S1. Characterization of the synthesized silver nanoparticles via **(A)** UV-Vis spectrum analysis, **(B)** Scanning electron microscopy (SEM), and **(C)** Dynamic light scattering (DLS).

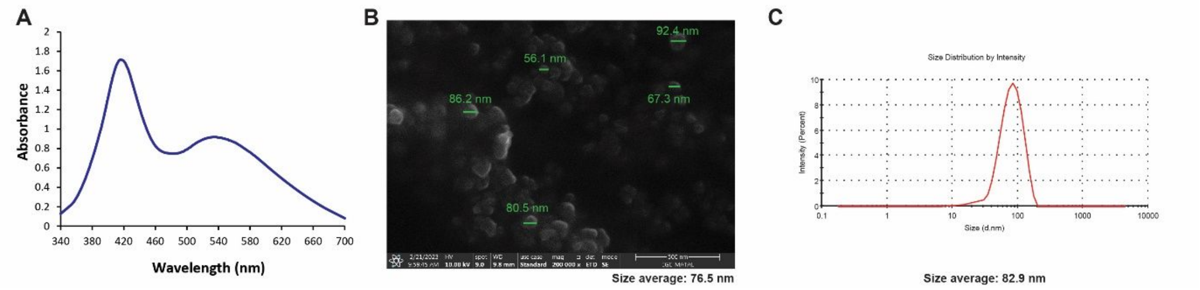

Figure S2. Optimization of the LP-µPAD with regards to sample volume, baking time, filter paper porosity, channel width. The pictures are of front and back view of the system. The sample employed is a red food dye.

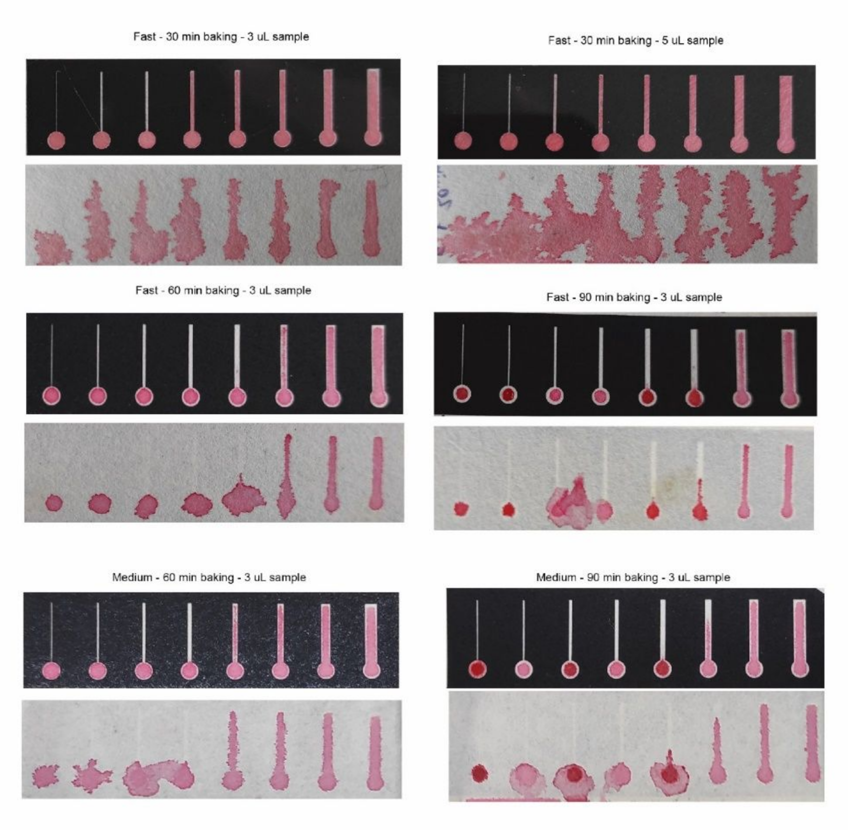

Supplement: Supplementary file 1 — ao3c04387_si_001.pdf [file ao3c04387_si_001.pdf]
